# Long-term Outcomes of Collagen Crosslinking for Early Keratoconus

**DOI:** 10.18502/jovr.v16i2.9077

**Published:** 2021-04-29

**Authors:** Akbar Derakhshan, Javad Heravian, Milad Abdolahian, Shahram Bamdad

**Affiliations:** ^1^Cornea Research Center, Mashhad University of Medical Sciences, Mashhad, Iran; ^2^Khatam-Al-Anbia Hospital, Mashhad University of Medical Sciences, Mashhad, Iran; ^3^Refractive Errors Research Center, Mashhad University of Medical Sciences, Mashhad, Iran; ^4^Department of Optometry, School of Paramedical Science, Mashhad University of Medical Sciences, Mashhad, Iran; ^5^Poostchi Ophthalmology Research Center, Shiraz University of Medical Sciences, Shiraz, Iran

**Keywords:** Cornea, Collagen Crosslinking, Keratoconus

## Abstract

**Purpose:**

To evaluate the long-term outcomes of collagen crosslinking in early keratoconus.

**Methods:**

Thirty eyes of twenty patients with early keratoconus were enrolled. Uncorrected visual acuity (UCVA), best spectacle corrected visual acuity (BSCVA), objective refraction, subjective refraction, corneal topography and pachymetry were assessed before and 3, 6, 12 months and 9 years after performing collagen crosslinking surgery.

**Results:**

The patients' mean age was 31.2 ± 5.59 years at nine-year follow-up (range, 25–44 years). The means of preoperative UCVA and BSCVA were 0.57 ± 0.34 and 0.15 ± 0.12 logMAR, respectively, and these values remained stable at the final follow-up (*P* = 0.990 and *P* = 0.227, respectively). The mean objective spherical equivalent decreased considerably from –6.00 ± 4.05 D preoperatively to –5.22 ± 3.71 D at the final follow-up (*P* < 0.05). The mean subjective spherical equivalent was –4.25 ± 2.87 D preoperatively and this value was stable at the last follow-up (*P* = 0.92). No considerable difference was found between the post- and preoperative mean objective cylinder values (*P* = 0.34). The mean subjective cylinder value changed significantly from –4.05 ± 1.85 D preoperatively to –3.1 ± 1.42 D at the final follow-up (*P* < 0.05). The mean central corneal thickness was 496.97 ± 45.95 µm preoperatively and this value was stable at nine-year follow-up (*P* = 0.183). No significant difference was found between the pre- and postoperative mean maximum and mean minimum corneal curvature values (*P* = 0.429 and *P* = 0.248, respectively). There were no significant postoperative complications.

**Conclusion:**

Corneal crosslinking in early keratoconus seems to be a safe procedure that can effectively stabilize UCVA, BSCVA, subjective SE and CCT, while improving objective spherical equivalent.

##  INTRODUCTION

Keratoconus is a bilateral, progressive, asymmetric, noninflammatory corneal ectasia. The cornea presumes a conical form due to its biomechanical instability leading to irregular astigmatism and reduction in visual quality. Treatment options available for increasing the visual acuity or/and halting the progression of keratoconus consist of spectacles, rigid gas permeable contact lenses,^[1]^ collagen crosslinking,^[2]^ intracorneal rings,^[3]^ and keratoplasty.^[4,5]^ Corneal collagen crosslinking (CXL) has been introduced as a promising method for keratoconus management. It was frequently reported that CXL could effectively stabilize the keratoconus progression, with a good safety profile.^[6,7,8]^

In 2003, Wollensak et al pioneered CXL treatment post-op progression of keratoconus. In CXL, the interaction between the riboflavin and ultraviolet-A (UVA, 365 nm) results in crosslinking between the intracellular matrix and collagen of the stroma, overwhelmingly in the anterior 300 µm, leading to enhanced strength of the cornea.^[8]^ Studies have indicated that collagen crosslinking leads to an increase of collagen fiber diameter^[10]^ and improves biomechanical stiffness^[11]^ by inducing increased covalent bond formation within or between collagen fibers in the corneal stroma.^[12]^ Some studies have demonstrated improvement in visual acuity,^[9,13,14,15]^ apical curvature of the cornea,^[9,16,17,18]^ contrast sensitivity improvement,^[19,20]^ and a decrease in refractive error.^[9,16,17,21]^ However, most of the studies have short follow-up period; therefore, this study aimed to assess the long-standing outcomes of CXL for early keratoconus.

##  METHODS

Our research was approved by Khatam al Anbia Hospital affiliated to Mashhad University of Medical Sciences. All steps of this study were based on the principles of the Declaration of Helsinki, and an informed consent was obtained from each subject after explaining the goals of the study. In this hospital-based prospective study, 32 eyes of 22 patients with early keratoconus were initially enrolled but 2 of them missed the follow-ups; therefore, we removed their data from this study. The diagnosis was performed based on video keratographic findings and all patients demonstrated progression before the surgery by longitudinal evaluation using corneal topography. The indications of keratoconus progression included an increase of 1.00 D or more in the cylindrical component of the manifest refraction, an increase of 1.00 D or more in the maximum corneal curvature, an increase of 0.50 D or more in the spherical equivalent (SE) manifest refraction in one year and a decrease of ≥5% in the central corneal thickness in three consecutive topographies in six months. Pre- and postoperative evaluation after 3, 6, and 12 months and then 9 years of follow-up consisted of uncorrected visual acuity (UCVA) and best spectacle corrected visual acuity (BSCVA) measurement, ultrasonic pachymetry (Tomey, Erlangen, Germany), corneal computerized topography (Technomed, Baseweile, Germany), and slit lamp and fundus examinations. Corneal thickness <400 μm, herpetic keratitis history, and concurrent infectious or autoimmune disease were the exclusion criteria. All procedures were carried out by the same surgeon (AD) in our institute under sterile conditions. For performing the surgery, corneal epithelium was removed by mechanical debridement over 9.0 mm of the central region of the cornea following administration of the topical anesthesia. Then, the photosensitizing solution (0.1% riboflavin within 20% dextran) was instilled every 3 min for 30 min, after epithelial debridement following topical anesthesia and inserting a wire lid speculum. Riboflavin penetrated into the anterior chamber and corneal stroma completely and the penetration was checked by slit lamp examination. Then, the UVA was irradiated on the cornea for 30 min (radiance of 3 mW/cm${}^{2}$), utilizing a 370 nm UVA double-diode light source. Over irradiating, the riboflavin solution was dropped every 5 min, and balanced salt solution was frequently applied intraoperatively to prevent dehydration of the cornea. Topical antibiotics were prescribed for five days along with tear substitutes for three to four weeks.

Data were analyzed using the SPSS.21 software (SPSS Inc., Chicago, Illinois, USA). Normality of the data was assessed using the Kolmogorov–Smirnov test. Comparisons were made using paired sample *t*-test. In all tests, *p*-values < 0.05 were considered significant.

##  RESULTS

### Vision Outcomes

**Figure 1:**
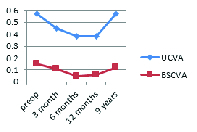
Stability of logMAR UCVA and logMAR BSCVA after corneal CXL.

The mean age of patients was 31.2 ± 5.59 years at the nine-year follow-up period. The mean UCVA was 0.57 ± 0.34 logMAR preoperatively and it did not significantly change at the final follow-up examination (*P* = 0.990). No significant difference existed between the post- and preoperative mean BSCVA (*P* = 0.227). At the last follow-up, BSCVA improved at least one Snellen line in 11 eyes (36.66%) and remained stable in 9 eyes (30%); 10 eyes (33.33%) lost one line or more. The pre- and postoperative values are represented in Table 1. Figure 1 shows the UCVA and BSCVA stability following corneal CXL.

**Figure 2:**
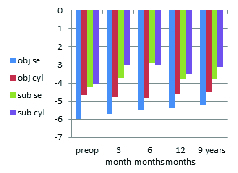
Changes in objective and subjective refraction (diopter).

**Figure 3:**
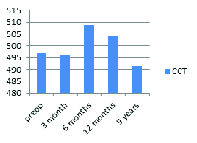
Changes in CCT (µm).

**Table 1:** Preoperative and postoperative patient data


**Parameters**	**Preoperative**	**3-months postoperative**	**6-months postoperative**	**12-months postoperative**	**9-years postoperative**
LogMAR UCVA P	0.57±0.34	0.45±0.34 *P* < 0.05	0.38 ±0.32 *P* < 0.05	0.38±0.33 *P* < 0.001	0.57 ±0.37 *P* = 0.990
LogMAR BCVA P	0.15±0.12	0.11±0.10 *P* < 0.05	0.05 ±0.06 *P* < 0.05	0.06±0.07 *P* < 0.05	0.12 ±0.12 *P* = 0.227
OBJ SE P	-6.00±4.0	-5.71±3.79 *P* = 0.09	–5.46 ± 3.44 *P* < 0.05	-5.40±3.44 *P* < 0.05	*–5.22* ± 3.71 *P* < 0.05
OBJ AST P	-4.68±2.28	-4.75±2.36 *P* = 0.37	–4.85 ± 2.03 *P* = 0.63	-4.64±2.16 *P* = 0.42	*–4.50* ± 2.06 *P* *= 0.34*
SUB SE P	-4.25 ± 2.87	-3.72±3.01 *P* = 0.055	–2.88 ± 2.23 *P* < 0.001	-3.75±2.83 *P* = 0.001	–3.8 ± 3.06 *P* = 0.09
SUB AST P	-4.05±1.85	-3.04±1.38 *P* < 0.05	–3.01 ± 1.48 *P* < 0.001	-3.52±1.70 *P* < 0.05	–3.1 ± 1.42 *P* < 0.05
CCT p	496.97 ± 45.95	496 ± 17.10 *P* = 0.21	508.88 ± 18.44 *P* = 0.52	504.20 ± 26.18 *P* = 0.07	491.43 ± 37.98 *P* = 0.18
K-max p	51.92 ± 5.47	54.71 ± 6.16 *P* = 0.23	48.06 ± 1.30 *P* = 0.45	50.95 ± 4.46 *P* = 0.23	51.40 ± 4.40 *P* = 0.42
K-min p	46.63 ± 4.36	43.55 ± 3.25 *P* = 0.477	44.91 ± 1.93 *P* = 0.18	45.71 ± 4.05 *P* = 0.85	46.34 ± 4.56 *P* = 0.24
UCVA, uncorrected visual acuity; BCVA, best corrected visual acuity; LogMAR, logarithm of the minimum angle of resolution; OBJ, objective; SUB, subjective; SE, spherical equivalent; AST, astigmatism; CCT, central corneal thicknes					

### Refractive Results

The mean objective SE improved significantly from –6.00 ± 4.05 D preoperatively to –5.22 ± 3.71 D (*P* < 0.05) at the nine-year follow-up. No significant difference was found between the post- and preoperative mean subjective SE (*P* = 0.92) and mean objective cylinder value (*P* = 0.348). The mean subjective cylinder value significantly changed from –4.05 ± 1.85 D preoperatively to –3.1 ± 1.42 D at the nine-year follow-up (*P* = 0.002). Figure 2 shows the changes in objective and subjective refraction.

### Central Corneal Thickness

No significant difference was found between the preoperative and postoperative mean central corneal thickness (*P* = 0.183). Figure 3 shows the changes in the central corneal thickness.

### Topographic Outcomes

No statistically significant difference was detected in the mean maximum (*P* = 0.429) and mean minimum corneal curvature (*P* = 0.248) at the nine-year follow-up. During the follow-ups, no macular and corneal abnormalities were observed.

None of the cases underwent repeated CXL.

##  DISCUSSION

Collagen crosslinking with riboflavin and UVA is a surgical technique used in the treatment of keratoconus. CXL is a surgical method utilized to improve the corneal rigidity, stabilize the corneal ectasia, and inhibit the progression of the keratoconus.^[5]^ Previous studies reported that collagen crosslinking improved visual, refractive, topographic and aberrometric values.^[15,16,21,22,23,24,25]^ However, some challenges are associated with the long-term outcomes of CXL.^[18,21,23,26,27,28]^ Vinciguerra et al^[26]^ reported that UCVA and BSCVA significantly increased two years following CXL. Raiskup-Wolf et al^[23]^ demonstrated stabilization and improvement of the cornea during long-term period following CXL. Keratoconus stability in 44 eyes after a minimum follow-up of 48 months was reported by Caporossi et al.^[21]^ O'Brart et al^[27]^ also showed that CXL was a safe and effective method used to stabilize the progression of the keratoconus over a long-term period. Hashemi et al^[28]^ reported halting of keratoconus progression up to five years of follow-up, while Wittig-Silva et al^[18]^ indicated improvements in maximum corneal curvature, UCVA, and BSCVA over a three-year follow-up period.

In our study, the stability of subjective SE was demonstrated similar to the reports by Wittig-Silva et al^[29]^ and Grewal et al.^[30]^ However, Caporossi et al,^[31]^ Wollensak et al,^[9]^ and Vinciguerra et al^[26]^ reported a decrease in subjective SE. In this study, the subjective SE and subjective astigmatism reduced significantly during the six-month follow-up and then gradually returned to the preoperative values. Significant changes in the cylinder values were reported at the first-year follow-up in some studies.^[32,33,34]^ Given the visual outcomes of the subjects in this study, there was no significant difference between pre- and postoperative values of UCVA (*P* = 0.990) and BSCVA (*P* = 0.227). Our results showed that 40% of patients had improved UCVA and 40% had improved BSCVA, whereas only 43.33% lost lines of UCVA and 23.33% lost BCVA at the last follow-up. UCVA and BSCVA increased significantly in the first six months due to reduction in refractive error and corneal steepening. Caporossi et al^[31]^ proposed that a decrease in the coma aberration following morphologic symmetry leads to an increase in BCVA. In our study, UCVA and BSCVA gradually returned to the preoperative values between the 6^th^ and 12^th^ postoperative months. At the final follow-up, the UCVA and BSCVA did not change significantly compared with the preoperative values. Caporossi et al^[35]^ reported mean increases of +0.12 and +0.10 Snellen lines in UCVA and BCVA 48 months after CXL, respectively. In our research, 30 of 32 cases were followed-up for nine years, while only 11 of 286 eyes included in their study had completed visits during four years. A 96% drop in the follow-up could lead to different results. Similarly, a study by Raiskup-Wolf et al^[23]^ revealed that only 5 out of 241 included eyes stayed in the study at six-year follow-up. One-year researches indicated that UCVA tended to increase during the first year after CXL.^[32,33,36]^ However, Asri et al reported no statistically significant changes.^[37]^ Our results also showed that BSCVA did not change significantly at the last follow-up. Some other researches demonstrated different results ranging from no change in BCVA at one-year follow-up^[37]^ to 1.26 Snellen lines^[9]^ and 0.1133 and 0.1832 logMAR increase in BCVA after CXL.

In this study, there were no statistically significant changes in CCT at any postoperative intervals. However, corneal thinning was reported in some studies.^[28,38]^ Greenstein et al^[38]^ showed corneal thinning and return to the standard values during the first three months and the first one year after treatment, respectively. An early reduction in CCT was also reported by Hashemi et al^[28]^ at the first postoperative month after an increment and achieving a plateau in this period; no alteration was reported following one and over five postoperative years. Caporossi et al^[21]^ and O'Brart et al^[27]^ reported that CT did not change significantly after long-term follow-up. Raiskup-Wolf et al^[23]^ showed an increase in CCT in the second year after CXL.

In our study, the steepest and flattest corneal curvatures following CXL did not change significantly at any interval after treatment. Our topographic results showed a mean decrease of 2.42 D in 56.6% of patients and a mean increase of 1.93 D in 43.33% of patients in the steepest corneal curvature at the last follow-up. A mean decrease of 2.01 D was reported by Wollensak et al in the maximum curvature values after four years.^[9]^ A mean reduction of 2.10 D in minimum corneal curvature values after six months were reported by Caporossi et al.^[31]^ Raiskup-Wolf et al^[23]^ reported 2.68 D, 2.21 D, and 4.84 D reduction of corneal curvature in the first, second, and third years after CXL, respectively. Steepest corneal curvature was reported as a weak parameter for both efficacy of the CXL and the keratoconus progression.^[40]^ The reason is that the steepest curvature characterizes the steepest curvature of the anterior corneal surface taken from a little region and it is not able to recognize the degree of ectasia; hence, keratoconus can progress without any change in the steepest corneal curvature.^[41]^

In conclusion, we recommend the use of CXL for patients with early keratoconus. Our findings indicate that the CXL procedure is an effective and a safe method for the treatment of keratoconus within a long-term postoperative follow-up duration. However, more studies with larger sample size are required to confirm the effectiveness of CXL.

##  Financial Support and Sponsorship

Nil.

##  Conflicts of Interest

None declared.
